# Morphological and functional differentiation in BE(2)-M17 human neuroblastoma cells by treatment with *Trans*-retinoic acid

**DOI:** 10.1186/1471-2202-14-49

**Published:** 2013-04-18

**Authors:** Devon Andres, Brian M Keyser, John Petrali, Betty Benton, Kyle S Hubbard, Patrick M McNutt, Radharaman Ray

**Affiliations:** 1 Research Division, US Army Medical Research Institute of Chemical Defense, 3100 Ricketts Point Road, Aberdeen Proving Ground, Maryland, 21010-5400, USA

**Keywords:** Neurons, M17, Neurotoxicity, Cell maturation, Differentiation, Retinoic acid, Neuroexocytosis, Voltage-gated calcium channels

## Abstract

**Background:**

Immortalized neuronal cell lines can be induced to differentiate into more mature neurons by adding specific compounds or growth factors to the culture medium. This property makes neuronal cell lines attractive as *in vitro* cell models to study neuronal functions and neurotoxicity. The clonal human neuroblastoma BE(2)-M17 cell line is known to differentiate into a more prominent neuronal cell type by treatment with trans-retinoic acid. However, there is a lack of information on the morphological and functional aspects of these differentiated cells.

**Results:**

We studied the effects of trans-retinoic acid treatment on (a) some differentiation marker proteins, (b) types of voltage-gated calcium (Ca^2+^) channels and (c) Ca^2+^-dependent neurotransmitter ([^3^H] glycine) release in cultured BE(2)-M17 cells. Cells treated with 10 μM *trans*-retinoic acid (RA) for 72 hrs exhibited marked changes in morphology to include neurite extensions; presence of P/Q, N and T-type voltage-gated Ca^2+^ channels; and expression of neuron specific enolase (NSE), synaptosomal-associated protein 25 (SNAP-25), nicotinic acetylcholine receptor α7 (nAChR-α7) and other neuronal markers. Moreover, retinoic acid treated cells had a significant increase in evoked Ca^2+^-dependent neurotransmitter release capacity. In toxicity studies of the toxic gas, phosgene (CG), that differentiation of M17 cells with RA was required to see the changes in intracellular free Ca^2+^ concentrations following exposure to CG.

**Conclusion:**

Taken together, retinoic acid treated cells had improved morphological features as well as neuronal characteristics and functions; thus, these retinoic acid differentiated BE(2)-M17 cells may serve as a better neuronal model to study neurobiology and/or neurotoxicity.

## Background

Neurotoxic chemicals, such as lead (Pb) and organophosphorus (OP) insecticides are prevalent in the environment. The use of different in vitro cell culture assays for predicting the *in vivo* effects of these chemicals have been extensively reviewed in recent years and the issues pertaining to their use have also been discussed [1-5]. The in vitro systems have been developed and utilized not only to understand the mechanisms of toxicity at the molecular and cellular levels but also to screen potential neurotoxicants. Potentially toxic compounds would be candidates for *in vivo* testing. The objective of neurotoxicologic studies on cells and tissues *in vitro* is to characterize the cellular and molecular substrates and pathways that contribute to impaired behavior, altered function, or pathological changes in the whole animal following exposure to a toxicant [1]. The two main types of cell culture systems used for *in vitro* neurological testing are (a) primary neuronal cell cultures dissociated from peripheral or central nervous system tissues and (b) clonal cell lines derived from tumors of neurological origin [2].

Primary neuronal cultures retain morphological, neurochemical, and electrophysiological properties of neurons *in situ*[2]. However, long-term culturing of primary neurons has been a major challenge. This creates difficulties in addressing the fundamental questions concerning cellular and molecular interactions among the many functionally distinct neuronal cell types that contribute to the development and functioning of the mammalian central nervous system [6]. Neuroblastoma cell lines have been used extensively as in vitro models for studies on neuronal development, neurological diseases and disorders as well as mechanisms of actions and neurotoxicity of compounds affecting the nervous system [2,7,8]. These *in vitro* models can provide a well-controlled system in which to study many of the critical cellular processes of neuronal development including proliferation, differentiation, growth, and synaptogenesis. Furthermore, cultured cell lines allow subtle changes in cell number, morphology, and functions to be readily detected compared to *in vivo* approaches and provide reproducibility in test results as well as providing a reduction in time, cost, and animal use [2,7].

Neuroblastoma cells can be differentiated by treatment with chemical agents into distinct morphologic cell types. These differentiated cells may be of different types: (a) substrate-adherent (S), which resemble non-neuronal precursor cells; (b) a sympathoadrenal neuroblastic (N); or (c) intermediate (I), which share elements of both S and N types [9]. Each of these cell types differs in their ability to induce a tumor. N-type cells are malignant, where as the S-type cells are not; however, the I-type cells show the greatest malignancy [10,11]. One common neuroblastoma cell type used for *in vitro* research is BE(2)-M17, commonly known and henceforth called M17, which is available from ATCC.

M17 is a human neuroblastoma cell line cloned from the SK-N-Be(2) neuroblastoma cell line isolated from a 2 year old male (ATCC, Manassas, VA). M17 cells are multipotential with regard to neuronal enzyme expression e.g., choline acetyltransferase, acetylcholinesterase and dopamine-β-hydroxylase implying cholinergic, dopaminergic and adrenergic properties. M17 cells convert glutamate to GABA [12], however, this property is much less than that exhibited by cerebellar cortex which contains GABAergic neurons [13]. There has been a great deal of research into differentiating the M17 cell line by treatment with *trans*-retinoic acid (RA); this treatment transformed the M17 cells to a morphologically distinct phenotype, i.e., neuroblastic with neuritic processes and with neurochemical characteristics [11,14]. This treatment was the forerunner to the current clinical therapy of neuroblastomas using isotretinoin. However, little research has been performed on the functional changes in M17 cells after exposure to RA.

In the present study, we differentiated M17 cells by treatment of cultures with 10 μM RA over a time period of 72 hours and investigated the morphological, neurochemical and functional changes that occurred. We observed the formation of neuronal processes and expression of proteins (including neuron specific enolase (NSE), synaptic vesicle associated protein – 25 kDa (SNAP-25), neurofilamentin heavy chain (NF-H) and medium chain (NF-M), synapsin, and nicotinic acetylcholine receptor alpha 7 (nAchR-α7); moreover, we measured two functional parameters i.e., voltage-gated Ca^2+^ channel activity and stimulus-induced Ca^2+^-dependent neurotransmitter ([^3^H] glycine) release. In our and other laboratories, Ca^2+^-dependent [^3^H] glycine release inhibition due neurotoxins (e.g. botulinum neurotoxins and tetanus toxin) has been shown to be a sensitive indicator of toxicity in neuronal models such as cultured primary mouse spinal cord cells [15-17] and synaptosomes prepared from rat brain and spinal cord [18]. We have also looked at the effects of RA differentiation on M17 cells toxicity studies of the known toxicant, phosgene (CG, COCl_2_). CG is a highly toxic chemical used in the manufacturing of pharmaceuticals, dyes, and polyfoam rubber products. CG causes bronchoconstriction, vasoconstriction and associated pathological effects that could be life threatening and is reported to have a neural component involved in its toxicity [19,20].

## Methods

### Cell culture

Frozen stock of the human neuroblastoma cell line, M17 (ATCC; Gaithersburg, MD), was cultured in 75 or 150 cm^2^ tissue culture flasks in a 1:1 mixture of Eagle’s Minimum Essential Medium with non-essential amino acids and F12 medium containing 10% fetal bovine serum inside a humidified cell culture incubator with 95% air plus 5% carbon dioxide according to company instructions to initiate the cultures. At 70-80% confluency, the monolayer cells were sub-cultured in appropriate vessels for use in experiments. When the cultures reached about 30% confluency, these cells were differentiated by treatment with 10 μM retinoic acid (Sigma, St. Louis, MO) added to the culture medium for 2 – 3 days or until they reached the desired confluency.

### Light microscopy

Cells were grown on coverslips to approximately 80% confluency. Cell media was decanted and the cells washed in 0.1 M sodium cacodylate buffer. The cells were then fixed in buffered 1.6% formaldehyde and 2.5% glutaraldehyde for 2–3 minutes at room temperature. The fixed cells were rinsed in 0.1 M sodium cacodylate buffer and stained with methylene blue in sodium borate solution for 1–2 minutes. The stained cells were rinsed with double distilled Millipore water. The cover slips were then inverted on a 1x3 positively charged slide. The cells were viewed and photographed at a magnification of 400X with an Olympus BX61 Microscope with NIKON photo assembly Digital Site DS-L1.

### Immunofluorescent staining

M17 cells were seeded onto 18 mm coverslips coated with poly-D-lysine (Sigma Aldrich). At described times, cells were fixed for 15 min in 3.7% formaldehyde, permeabilized with 0.1% saponin in PBS and blocked with 3% BSA (PBSS). Primary antibodies against the neuron-specific proteins β3-tubulin and synapsin-1/2 (Synaptic Systems, Gottingen, Germany) were diluted 1:1000 in PBSS and applied for 1 h at room temperature, followed by Alexa-conjugated secondary antibodies (Invitrogen) diluted 1:500 in PBSS. Coverslips were mounted onto slides with Prolong Gold DAPI (Invitrogen) and imaged using a Zeiss LSM 700 confocal microscope. Z-stack images were converted to a maximum projection image using Zen 2009 (Zeiss) software.

### SDS-PAGE and western blotting to assess levels of neuronal proteins

The level of neuronal proteins and SNAP-25 in M17 cells was assessed by SDS-PAGE and Western blotting following the method described by Ray et al. [21]. Briefly, cells harvested in ice-cold physiological saline were lysed by incubating with a lysis buffer containing 10 mM Tris–HCl, 150 mM NaCl, 1 mM EDTA, 1 mg/mL BSA, 1 mM PMSF, 1% Triton X-100 and protease inhibitors present in a protease inhibitor cocktail (cat. # P8340, Sigma, St. Louis, MO) which was included throughout the wash and solubilization steps to prevent protein degradation during the assay. The lysates were cleared by centrifugation at 16,000 x g for 10 min and immediately analyzed by SDS-PAGE. Each gel (8 – 16.5% acrylamide) lane was loaded with approximately 30 μg protein, which was determined by bicinchoninic acid (BCA) protein assay. Dry transfer to polyvinylidene fluoride (PVDF) membranes was performed using the iBlot™ System (Invitrogen, Carlsbad, CA) and blocked in 2% (w/v) bovine serum albumin for 1 hr (this and all subsequent incubations were performed in physiological saline, pH 7.5, room temperature). To determine the protein levels of specific neuronal proteins, blots were probed with primary antibodies overnight at 4°C. The primary antibodies include neuron specific enolase (NSE; N6049), SNAP-25 (59682), and vimentin (V2258), all purchased from Sigma (St. Louis, MO); M1 muscarinic acetylcholine receptor (M1 mAChR, AB5164), choline acetyltransferase (ChAT, AB144P) from Cell Signaling (Boston, MA); synapsin (AB1543), neurofilament medium (145kDa, AB1981), and neurofilament heavy (200 kDa, AB1989) from Millipore (Billerica, MA); and nicotinic acetylcholine receptor α7 (nAChR-α7, ab23832, Abcam, Cambridge, MA). Antibodies to β-Actin (4967) or GAPDH (2118), both from Cell Signaling (Boston, MA), were used for normalization to confirm equivalent gel loading. This was followed by one hour incubation with a 1:1000 dilution of polyclonal peroxidase-conjugated anti-mouse IgG followed by diaminobenzidine hydrogen peroxide (H_2_O_2_) and a 1:1000 dilution of appropriate peroxidase-conjugated anti-rabbit IgG. Detection was performed by fluorescent detection of enhanced chemifluorescent (ECF) substrate (Amersham, NJ) on the Typhoon+ (GE Healthcare, Piscataway, NJ). The relative amount of protein was quantified by densitometric analysis using Image J program (NIH public domain program, (http://rsbweb.nih.gov/ij/index.html) and normalized against the respective house- keeping protein (GAPDH or β-Actin) for each parameter studied. To ensure equal loading of proteins on the gel, each marker protein was normalized against a house-keeping protein (GAPDH or β-Actin). The house-keeping protein needed to be separate from the marker protein on the gel. Differences in marker proteins in differentiated vs. undifferentiated cells were assessed in terms of % normalized optical density in differentiated cells compared to undifferentiated (no RA) controls as shown in the table. Normalized optical density of undifferentiated control was 100% for each type of marker protein.

### Neurotransmitter release

High potassium-stimulated Ca^2+^-dependent neurotransmitter release was determined as previously described [22]. Cultures were incubated with 2 μCi/mL [^3^H] glycine for 30 min at 37°C to label a releasable [^3^H] glycine pool. Cells were then washed with a series of low K^+^ (3 mM)-containing isotonic buffers (136 mM NaCl, 0–2 mM CaCl_2_, 1 mM MgCl_2_, 10 mM HEPES, 10 mM glucose, and 0.1% BSA, ph 7.25, osmolarlity of 325 ± 5 mmol/kg). Unless otherwise indicated, [^3^H] glycine release was stimulated by addition of 80 mM K^+^ and 2 mM Ca^2+^ to cultures; stimulation medium was collected after 5 min at 37°C. Calcium-dependent release was determined by subtracting baseline radioactivity secreted from cultures in the absence of Ca^2+^, and expressed as a percentage of the total cellular radioactivity.

### Radiolabeled ^45^Ca^2+^ uptake due to KCl depolarization

M17 cells were seeded into 24 well plates (Corning, Lowell, MA) and cultured with 1 μCi/ml of [^3^H]-Valine (Perkin-Elmer, Waltham, MA) for 24 hours. The medium was then aspirated and cells were washed two times with 1 ml of a physiological balanced solution (PBS) containing in mM: 128 NaCl, 5.9 KCl, 1.28 CaCl_2_, 1.2 MgCl_2_, 17 HEPES, 3.3 glucose. All components for this solution were obtained from Sigma-Aldrich (St. Louis, MO). Cells were then incubated with an additional 1 ml aliquot of PBS for one hour for equilibration. The PBS was replaced with either PBS or stimulation medium containing 25 – 100 mM KCl and 1 μCi/ml ^45^CaCl_2_. The different KCl concentrations were adjusted by reducing the NaCl concentration proportionately to maintain iso osmolality. The addition of 1 μCi/ml of ^45^CaCl_2_ did not significantly alter the osmolality or the concentration of CaCl_2_ (data not shown). After 4 minutes, the radioactive medium was aspirated and ice-cold PBS was used to wash cells twice. The cells were then lyzed by adding 0.4 N NaOH solution and rocked overnight at 4°C. The next day, the 0.4N NaOH solution was pH neutralized with 0.4N HCl + Tris. The neutralized solution was added to scintillation fluid and ^45^Ca and ^3^H levels were measured on a Beckman LS6500 multi-purpose scintillation counter (Beckman-Coulter, Brea, CA). Calcium-dependent release was determined by subtracting baseline radioactivity secreted from cultures in the absence of Ca^2+^, and expressed as a percentage of the total cellular radioactivity. Total cellular radioactivity was calculated by adding the ^45^Ca^2+^ cpm released upon stimulation with high KCl and the ^45^Ca^2+^ cpm present in the resting medium with low KCl before and after stimulation.

### Direct Ca^2+^ uptake assay (Fluo-4 Direct™ assay kit)

The Ca^2+^ uptake assay kit (Fluo-4 Direct™, Invitrogen, Carlsbad, California) contains a fluo-4 analog that produces a large fluorescence intensity increase in response to Ca^2+^ binding with fluorescence excitation at 495 nm and emission at 516 nm. The Fluo-4™ Direct Ca^2+^ Assay kit was used according to the manufacturer’s instructions. Briefly, 2X Fluo-4 Direct Ca^2+^ assay buffer (1M HEPES in Hanks buffered saline solution, pH 7.3) was thawed and mixed with 5 mM probenecid. The assay was conducted in monolayer cultures. The 2X Fluo-4 Direct Ca^2+^ assay buffer with 5 mM probenecid was added in equal volume to the sample volume and incubated at 37°C for approximately one hour (Fluo-4 loading of cells). The fluorescence (absolute units) was measured using the SpectraMax Gemini EM (MDS Analytical Technologies, Toronto, Canada) using excitation and emission wavelengths mentioned above.

### Phosgene (CG) exposure

CULTEX air/liquid exposure system for either 12 mm or 24 mm culture filters was purchased from VetroCell (Germany). Prior to exposure to CG, the cells grown on these filters were transferred into the exposure device. Cells were nourished by culture growth medium below the filter membrane and exposed directly to either air (unexposed control samples) or air/CG (experimental samples) without growth medium on top of the cells. The vapor exposure system was moved to a chemical agent exposure hood and exposed to CG by connecting to the inlet of the CULTEX system that ends on a hyperboloid-shaped air distribution (trumpet) ensuring uniform exposure of the aerosol on the surface of the cell culture [23-27]. The outlet of the exposure system was passed through a decontamination solution. After exposure to air or CG, the cells were fed with fresh medium from the upper side and free intracellular Ca^2+^ was monitored by the Fluo-4 Direct Assay described above.

To assess intracellular free Ca^2+^ changes due to CG exposure, undifferentiated or differentiated M17 cells were first loaded with Fluo-4 and an initial fluorescence reading was taken to record the basal Ca^2+^ level (i.e., the resting intracellular Ca^2^+ levels without any treatment). The cells were then exposed to either 0 ppm (air) or 16 ppm of CG (in air) for 8 min at a flow rate of 8.13 ml/well/min. The free intracellular Ca^2+^ changes were monitored directly following exposure as a function of time.

### Treatment with Ca^2+^ ionophore A23187

The Ca^2+^ Ionophore A23187 (Invitrogen, Carlsbad, California) was prepared at stock concentration in DMSO according to manufacturer’s instructions. Further dilutions were prepared in M17 medium. A23187 was added directly to M17 cells cultured in transwell inserts to obtained final concentration of 5 μM. To assess intracellular free Ca^2+^, the cells were first loaded with Fluo-4 prior to addition of A23187 and the change in intracellular Ca^2+^ concentration was measured directly following addition of A23187 using Fluo-4 Direct Assay.

## Results

### Morphological changes and possible synaptic activity

**Figure 1:**
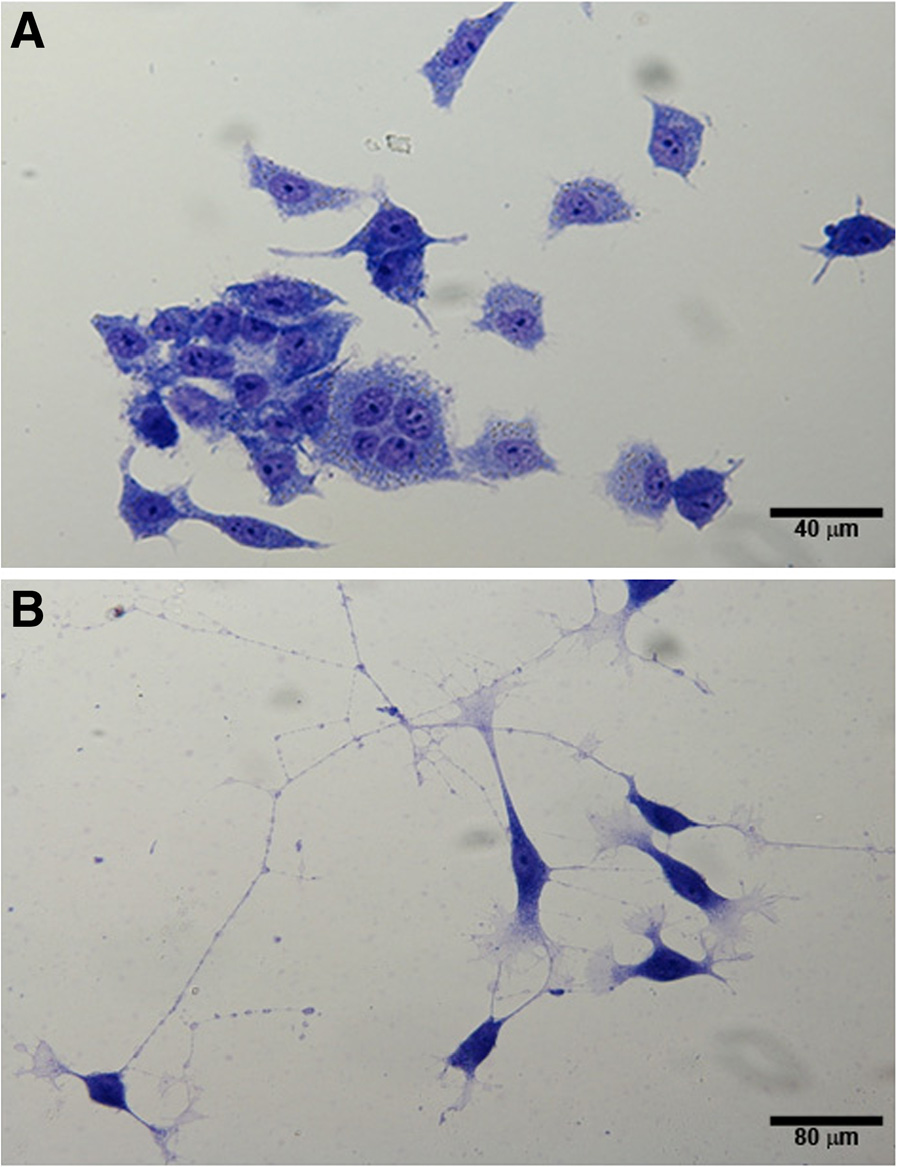
**Morphology of M17 cells without and with RA differentiation.** M17 neuroblastoma cells were grown on cover slips and treated with or without 10 μM RA for 72 hours to induce differentiation. Cells were fixed, stained, and light microscopy images were taken. (**A**) Undifferentiated cells, (**B**) Differentiated cells. 40X magnification; Scale bar is approximate.

To establish the conditions for RA treatment to induce cellular differentiation, we examined the effects of different RA concentrations (1, 5, and 10 μM) and times (48 – 120 hours). M17 cells transformed from an immature to a more mature neuronal state after 72 hours of exposure to 10 μM RA. Light photomicrographs of cells grown with or without 10 μM RA are shown in Figure 1. Undifferentiated cells had a more rounded morphology with few neurites or apparent synapses. Upon exposure to RA, the cells changed to a neuron-like triangular shape. Some of these cells were multinucleated with extended neurites. Treatment using less than either 10 μM RA or 72 hrs did not produce the morphological differentiation as shown as Figure 1 (data not shown). Moreover, functional indicators of neuronal differentiation as evidenced by immunofluorescence staining of synaptic marker proteins or stimulated neurotransmitter ([^3^H] glycine) release described below were either not prominent (morphology) or inconsistent (neuronal function) (data not shown). The treatment conditions that we selected were previously used to show early effects of RA-induced neuronal differentiation and oncogene (p26-Bcl-2) expression in another BE(2) variant cell clone [28].

**Figure 2:**
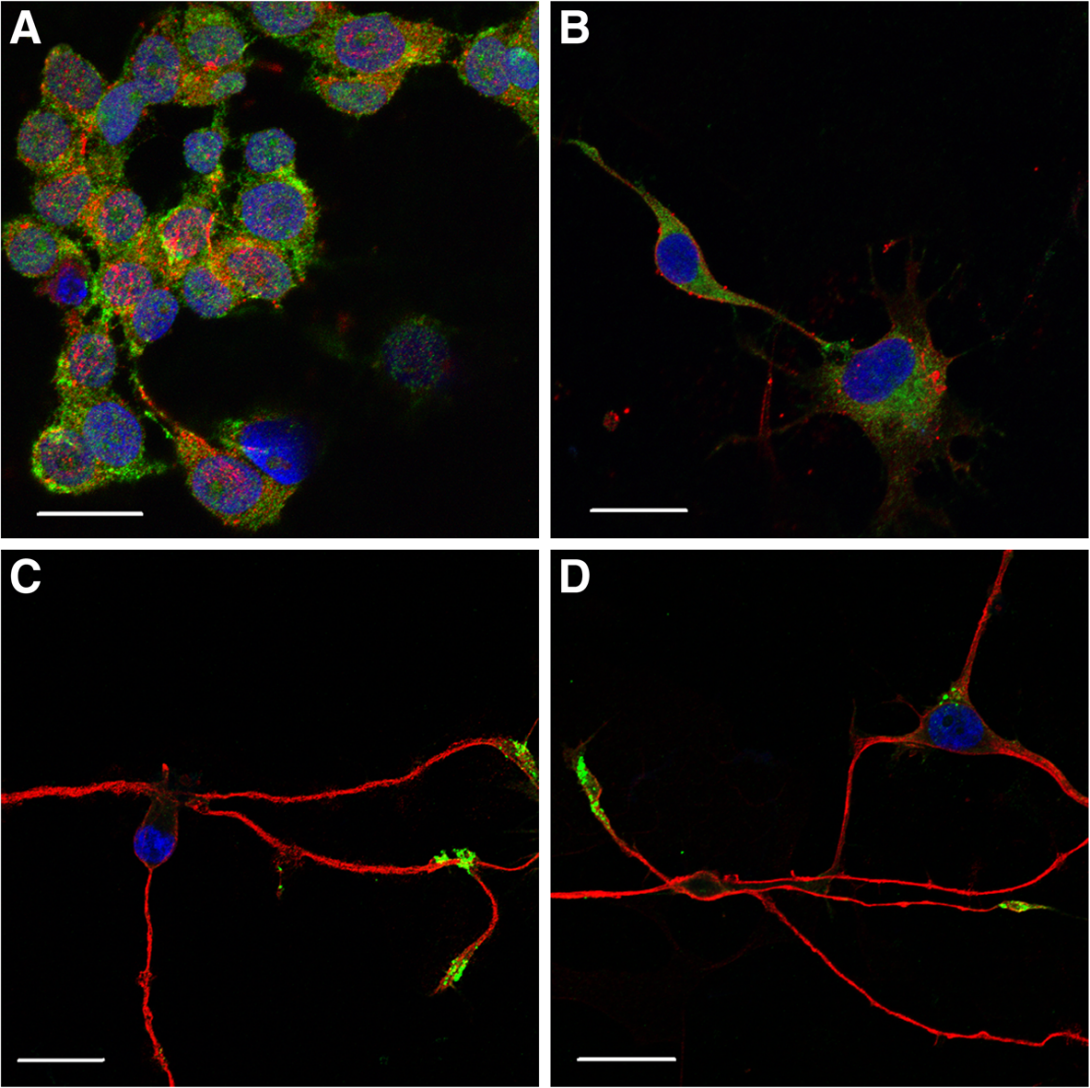
**Progressive development of neuronal morphologies induced by RA treatment.** M17 neuroblastoma cells were grown on cover slips. Cells were fixed, stained, and immunofluorescent images were taken (63X). β3-tubulin (red), synapsin-1/2 (green), and nuclei (blue). Undifferentiated M17 cells (**A**), development of radial glia-like morphology 72 h after RA addition (**B**), neurite extension and network formation (**C**, **D**).

**Figure 3:**
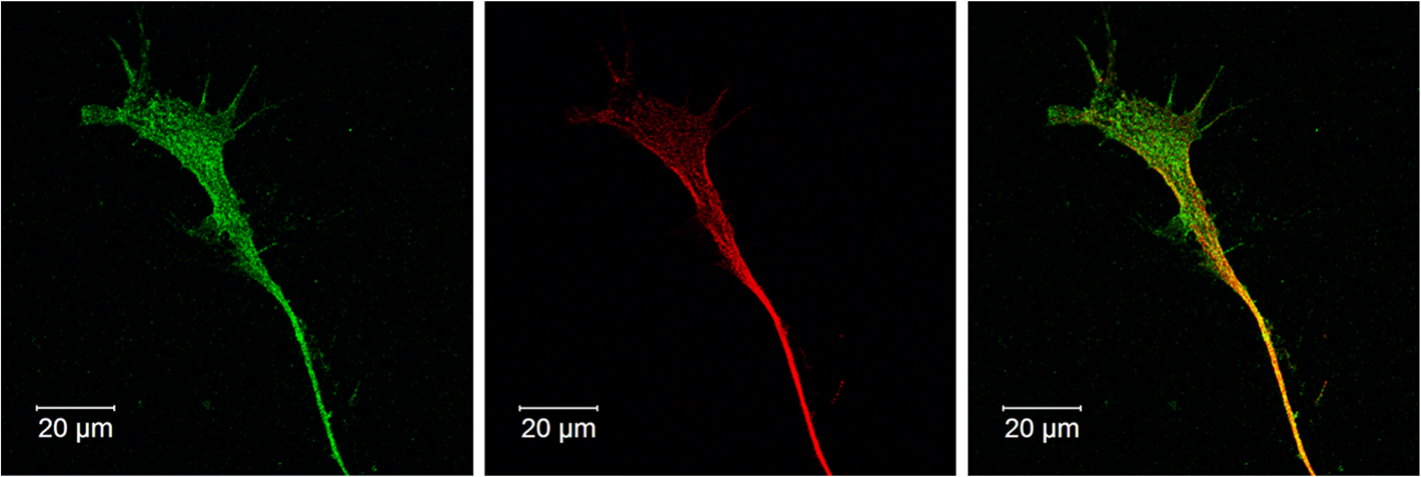
**Growth cone organization 120 h after RA treatment.** M17 neuroblastoma cells were grown on cover slips. Cells were fixed, stained, and immunofluorescent images were taken (63X). Synapsin-1/2 (green), β3-tubulin (red) and nuclei (blue). β3-tubulin expression is predominantly localized to the neurite body, whereas synapsin-1/2 accumulates within the growth cone.

To evaluate morphologic evidence of synaptogenesis, we used immunofluorescence staining to compare expression and localization of the pre-synaptic marker synapsin-1/2 and the early-stage neuron-specific marker β3-tubulin in undifferentiated versus differentiated M17 cells. As was observed using light microscopy (Figure 1), undifferentiated M17 cells were characterized by a rounded morphology, with few processes, and synapsin-1/2 and β3-tubulin were distributed throughout the cell body (Figure 2A). By 72 h after RA treatment (10 μM) some M17 cells had developed a radial glial-like morphology with bilateral processes, although compartmentalization of synapsin-1/2 and β3-tubulin was not observed (Figure 2B). By 120 h differentiated M17 cells exhibited extended processes with neuritic morphologies (Figure 2C, D). Synapsin-1/2 and β3-tubulin were observed to be diffusely present within the cell body (supporting Additional file 1: Figure S1), but also exhibited punctate localization along neurites (Figure 2C, D; supporting Additional file 1: Figure S1). β3-tubulin and synapsin-1/2 also exhibited distinct expression patterns within the neurite growth cone, such that β3-tubulin was concentrated along the neurite extension whereas synapsin-1/2 accumulated within the growth cone tip (Figure 3)*.*

### Expression of neuron specific proteins

**Figure 4:**
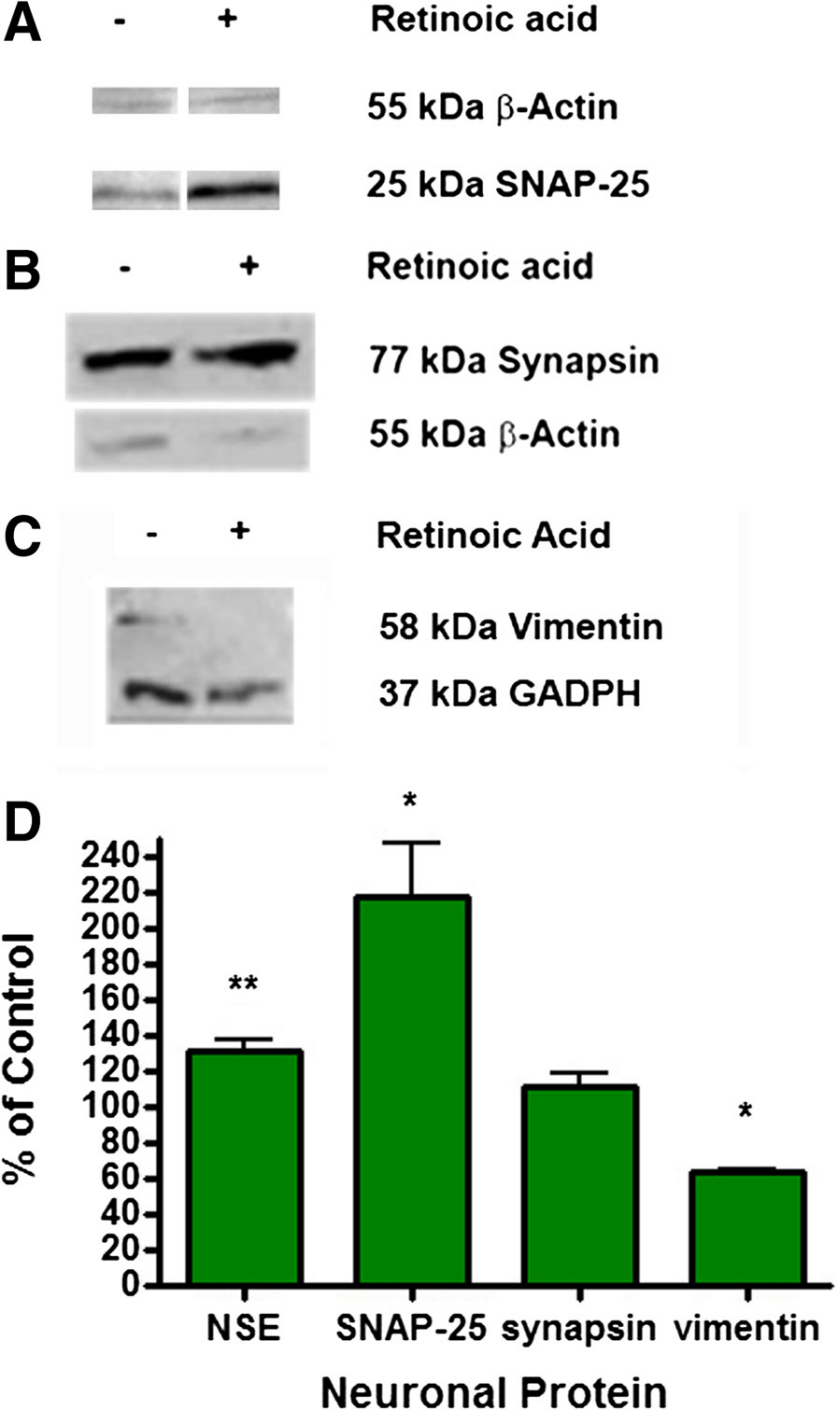
**Expression of Neuron Specific Proteins in M17 cell cultures.** M17 neuroblastoma cells were grown and treated with or without 10 μM RA for 72 hours to induce differentiation. The cells were washed and solubilized in sample buffer and analyzed by Western blotting for (**A**) SNAP-25, (**B**) synapsin, and (**C**) vimentin. Either β-actin or GAPDH was used as a house keeping protein marker to show equal protein loading of gels. The relative amount of each marker protein was quantified by densitometric analysis using Image J program (NIH public domain program, http://rsbweb.nih.gov/ij/index.html). Differences in marker proteins in differentiated vs. undifferentiated cells were assessed in terms of % normalized optical density in differentiated cells compared to undifferentiated (no RA) controls as shown in the bottom panel (**D**). Normalized optical density of undifferentiated control was 100% for each type of marker protein. **p<0.01, *p<0.05, using the student t test. n=6.

**Figure 5:**
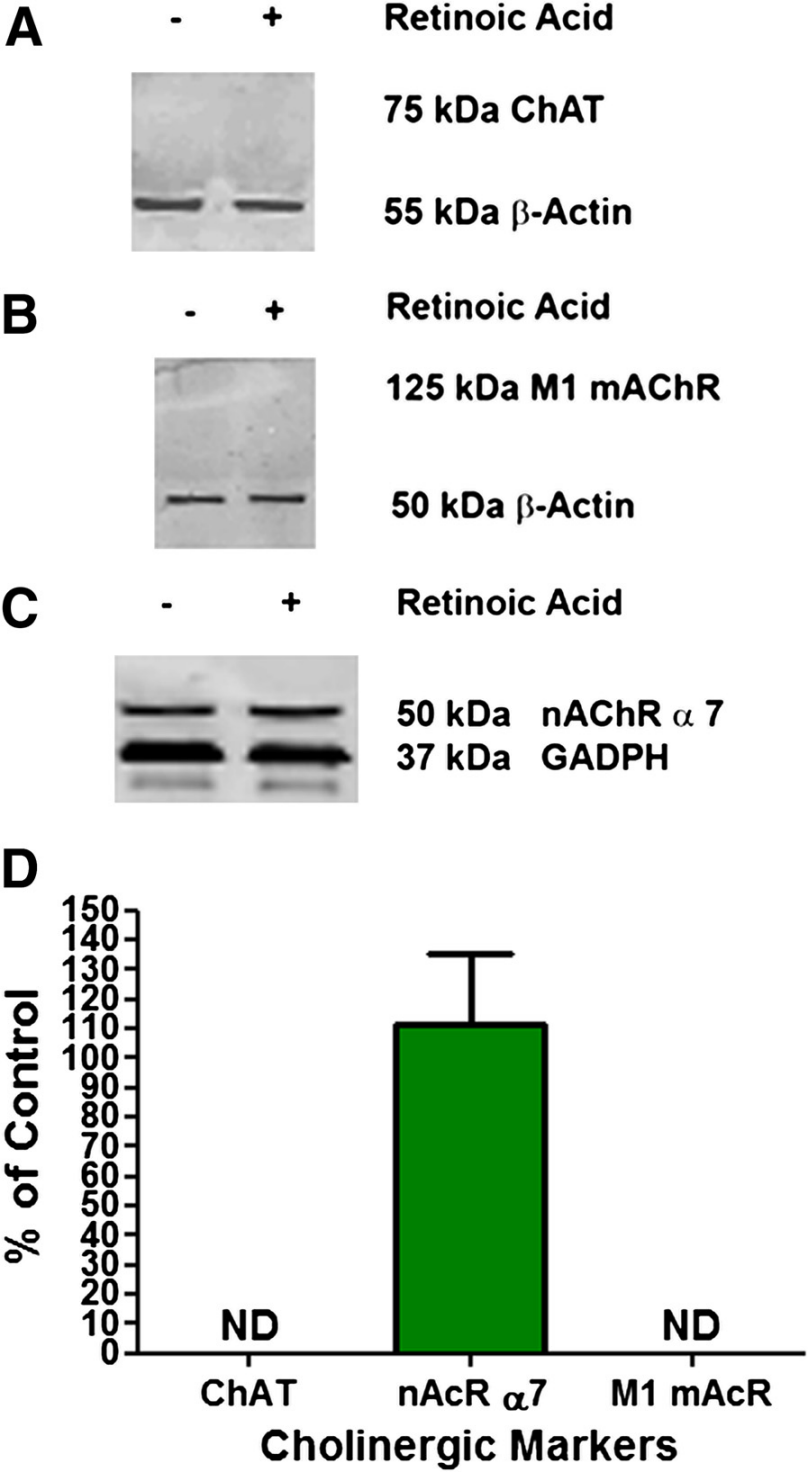
**Expression of markers specific for cholinergic neurons.** M17 cells were grown without or with 10 μM RA for 72 hours to induce differentiation. The cells were washed and solubilized in sample buffer and analyzed by Western blotting for (**A**) choline acetyltransferase (ChAT), (**B**) M1 muscarinic acetylcholine receptor (mAcR), and (**C**) nicotinic acetylcholine receptor α 7 (nAcR a-7). Either β-actin or GAPDH was used as a house keeping protein marker to show equal protein loading of gels. The relative amount of each marker protein was quantified by densitometric analysis using Image J program (NIH public domain program, http://rsbweb.nih.gov/ij/index.html). Differences in marker proteins in differentiated vs. undifferentiated cells were assessed in terms of % normalized optical density in differentiated cells compared to undifferentiated (no RA) controls as shown in the bottom panel (**D**). Normalized optical density of undifferentiated control was 100% for each type of marker protein. ND = not detected and as such could not be quantified. For M1 mAcR, the difference between undifferentiated vs differentiated was not statistically significant (student t test). n=4.

The levels of expression of selected neuron specific proteins were studied to help establish the degree of maturation of M17 cells with and without 10 μM RA treatment. Cultures of untreated and RA treated cells were harvested and cell lysates were used for Western blot analyses. The same lysates were used to detect NSE, SNAP-25, synapsin, neurofilaments M and H, nAChR, mAChR, and ChAT. The immunoreactictivity of selected neuronal proteins are shown in Figure 4. Each specific protein was identified by a single band marked at the appropriate molecular weight. Band intensities were quanitatively estimated and the results are shown as the of % normalized optical density in differentiated cells compared to undifferentiated (no RA) controls (Figures 4D and 5D). The levels of SNAP-25 (Figure 4A), synapsin (Figure 4B), and neurofilament M (data not shown) showed an increase in differentiated vs. undifferentiated cells. However, no neurofilament-light (NF-L) was detected (data not shown). The level of nAChR α7 (Figure 5C) remained the same upon differentiation with RA. Vimentin was expressed in undifferentiated cells but decreased upon differentiation (Figure 4C). Neither undifferentiated nor differentiated cells expressed any detectable amount of ChAT (Figure 5A) and M1 mAChR (Figure 5B). The specific antibodies used to detect ChAT and M1 mAChR as described above under Methods positively identifies these proteins in rat brain tissue (Hoard-Fruchey et al., unpublished results).

### Neurotransmitter release

**Figure 6:**
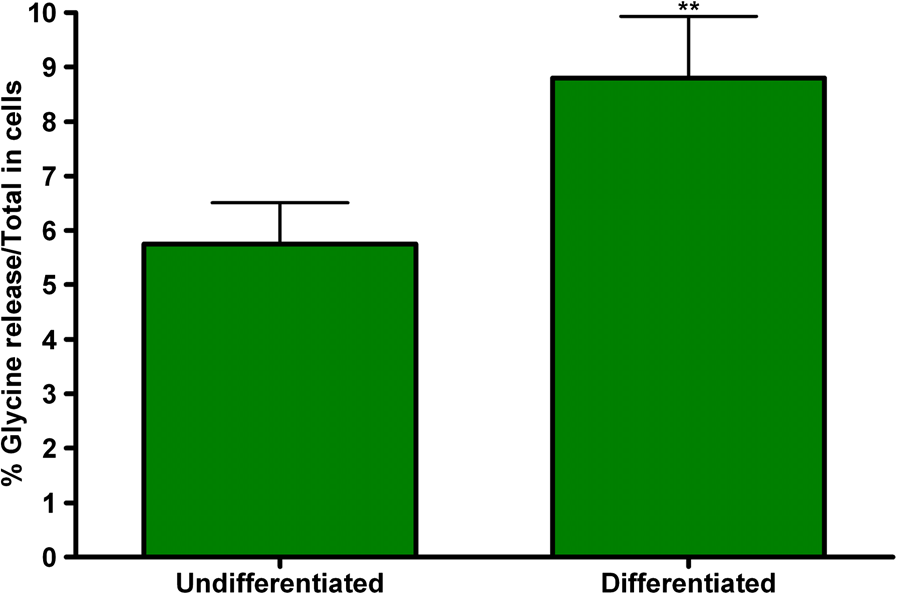
**Effect of 10 μM RA on K^+^-evoked [^3^H]-glycine release in M17 cells.** M17 neuroblastoma cells were grown and treated with or without 10 μM RA for 72 hours to induce differentiation. The treated M17 cells were then incubated with 2μCi/mL of [^3^H] glycine for 30 min and then stimulated with 80 mM KCl. The % glycine release/Total release was then calculated. ** Significantly different from control on corresponding day after Student’s t-test (p<0.05).

The effect of differentiation on functional neuroexocytosis was assessed in M17 cells by measuring potassium stimulated [^3^H] glycine release. Undifferentiated M17 cells had a basal level of stimulated glycine release; this was significantly increased in differentiated cells (Figure 6). This increase in neurotransmitter release correlates with the increase in the neuronal markers, particularly the vesicular fusion protein, SNAP-25.

### Expression of functional voltage-gated Ca ^2+^ channels

**Figure 7:**
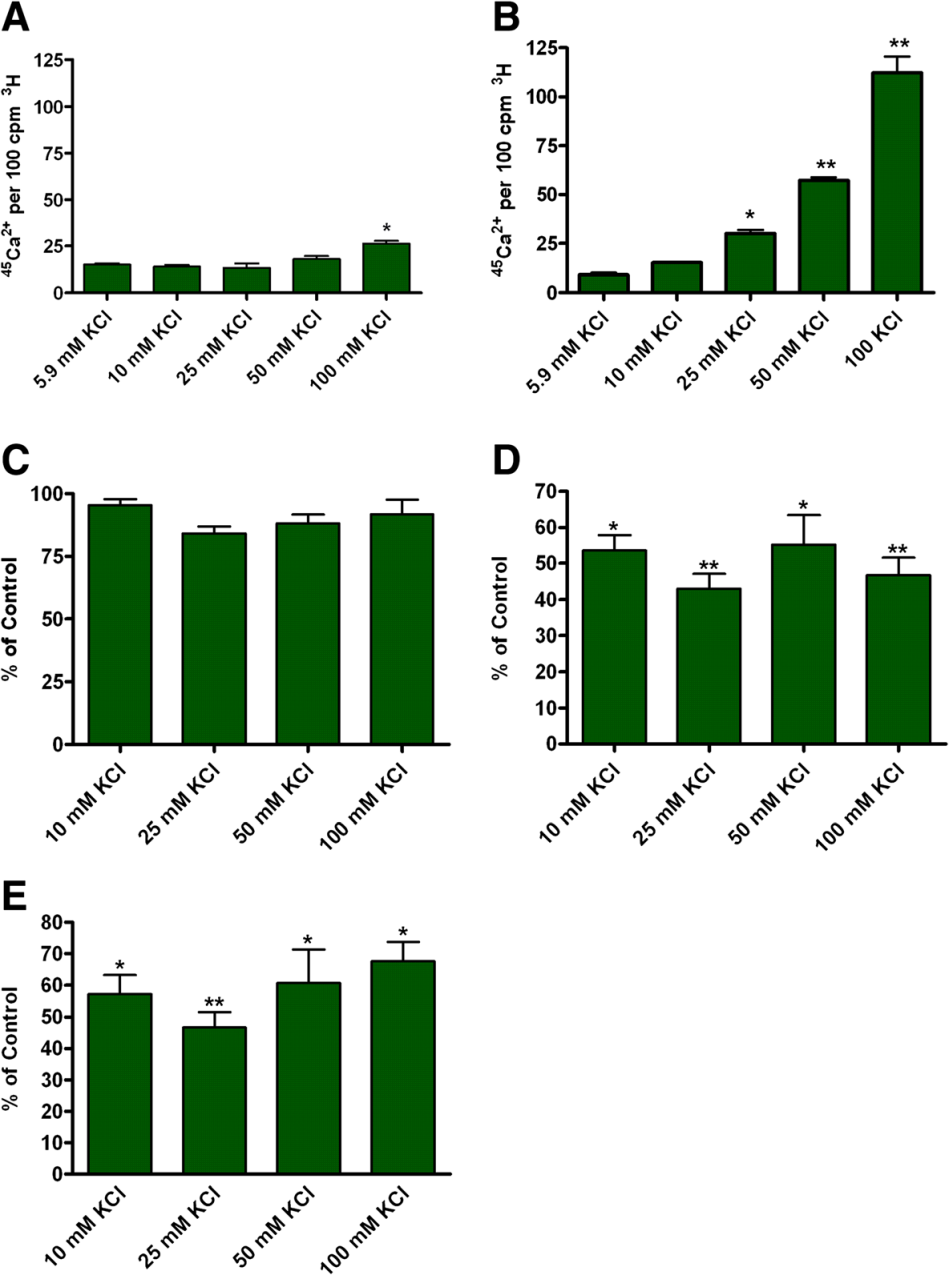
**Effect of 10 μM RA on the expression of functional voltage-sensitive.** Ca^2+^ channels in M17 cells. M17 cells were treated with 1 mCi/ml ^3^H-Valine 24 hours prior to each experiment. M17 cells were either (**A**) undifferentiated cells stimulated for 4 minutes with KCL, or differentiated cells (**B**) stimulated for 4 minutes with KCl, (**C**) KCl + 10 μM NNC 55-0396/KCl, (**D**) KCl + 1 mM w conotoxin, GVIA, or (E) KCl + 300 nM w agatoxin IVA. Each of these KCl solutions contained 1 mCi/ml of ^45^Ca^2+^. The ratio of ^45^Ca^2+^/^3^H was then used to calculate the percentage difference of Ca^2+^ channel activity. % of control was calculated by dividing the experimental ratio of ^45^Ca^2+^/^3^H by the ratio of ^45^Ca^2+^/^3^H generated with 5.9 mM KCl alone. n=4 *p<0.05 when compared to 5.9 mM KCl. **p<0.01 when compared to 5.9 mM KCl.

It is interesting to note that in M17 cells the increase in ^45^Ca^2+^ uptake due to increasing concentrations of KCl in the incubation medium was very pronounced (five-fold) in differentiated cells (Figure 7B); however, undifferentiated cells had a small increase (Figure 7A). To characterize the type of Ca^2+^ channels involved, ^45^Ca^2+^ uptake was measured in the absence and presence of known channel subtype specific antagonists. The antagonists were as follows: NNC 55–0396 (Ca_v_3.1 and 3.2); conotoxin GVIA (Ca_v_2.2); and agatoxin IVA (Ca_v_2.1). The concentrations of the antagonists used where according to published reports [29-31]. The results (Figure 7C-E) showed a small reduction in stimulated ^45^Ca^2+^ uptake due to NNC 55–0396 (10 μM), whereas a 50-60% reduction was observed due to conotoxin (1 mM) or agatoxin (300 nM). These observations suggested functional Ca_v_2.1 and 2.2 channels in differentiated cells.

### Differentiated M17 cells as a model to study neurotoxicity

**Figure 8:**
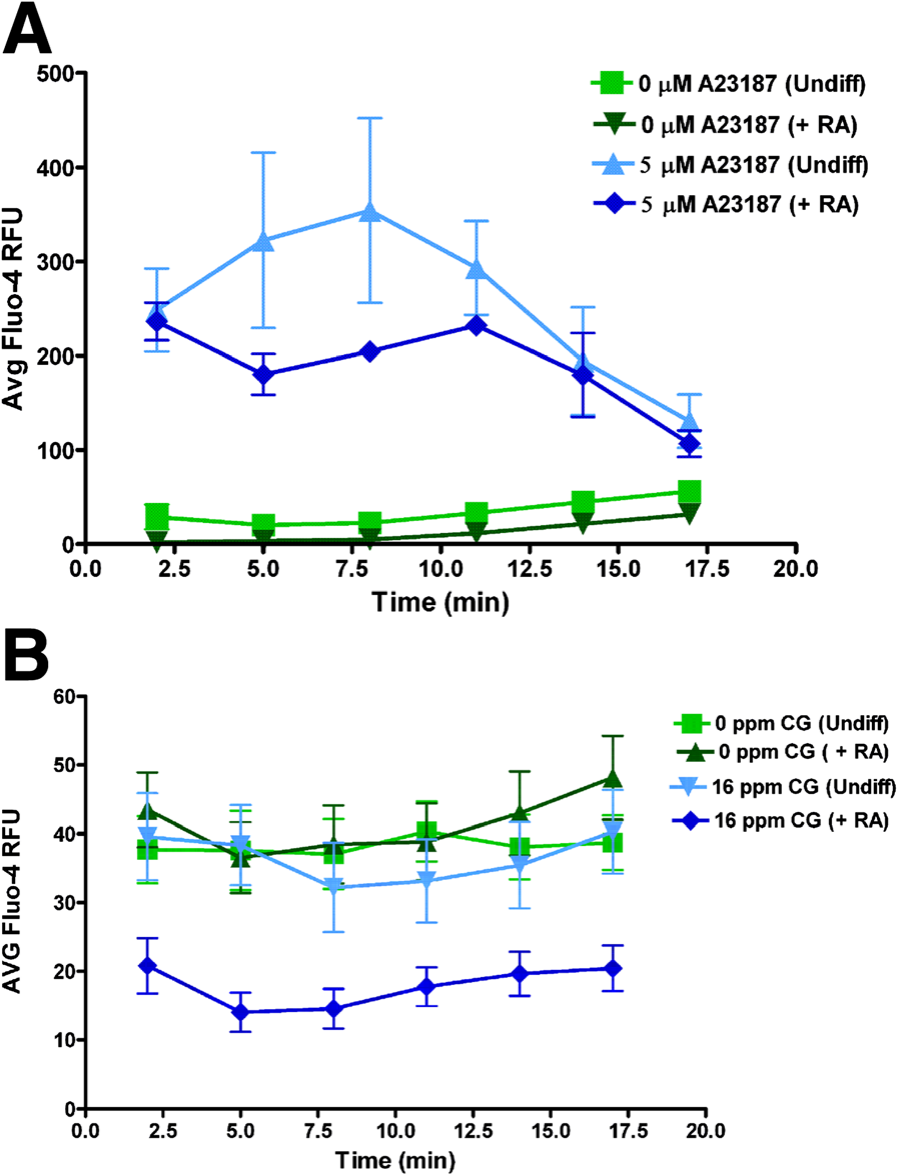
**The effects of a Ca^2+^ionophore and phosgene (CG) on intracellular Ca^2+^ changes in differentiated vs. undifferentiated M17 cells.** M17 cells were cultured in Transwell inserts without and with RA differentiation to 80% confluency. They were then exposed to 5 μM Ca^2+^ ionophore, A23187 or 0 and 16 ppm CG. Intracellular free Ca^2+^ levels were monitored using Fluo-4 Ca^2+^ indicator assay. (**A**) Change in intracellular Ca^2+^ following A23187 exposure (N=3); (**B**) Change in intracellular Ca^2+^ following either 0 ppm (air) or 16 ppm CG exposure for 8 min (N=13). *p<0.05 when compared to treated (A23187 or CG) cells.

We hypothesized that the lack of expression of voltage-gated Ca^2+^ channels in undifferentiated M17 cells limits their use as neurotoxicity model. To test this hypothesis, we studied the effect of a toxic industrial chemical, CG on intracellular free Ca^2+^ concentration ([Ca^2+^]_i_) in both undifferentiated and RA differentiated M17 cells. First, we tested the effect of the non-specific Ca^2+^ ionophore A23187 (5 μM) on [Ca^2+^]_i_. The results shown in Figure 8A demonstrated a large increase in [Ca^2+^]_i_ due to A23187 as expect without any significant difference between undifferentiated vs. differentiated cells. CG (16 ppm) caused significant decreases (p<0.05) at all times tested only in the RA differentiated cells without any effect in undifferentiated cells (Figure 8B).

## Discussion

Neuronal cell models have been tested for their use in predicting *in vivo* effects of different neurotoxic substances [1,2,4,5]. Attempts have been made to develop and to utilize these in vitro neuronal models to study the mechanisms of toxicity due to chemical and biological compounds at cellular and molecular levels. Moreover, these models have also been tested for their use in rapid screening of potential neurotoxicants out of which positive compounds would be selected for *in vivo* evaluation. Prior studies using *in vitro* cellular models were intended to generate preliminary mechanistic and toxicity information while reducing animal use and associated high cost of in vivo testing. The following are the three different types of *in vitro* cellular models primarily used in biomedical research; (1) primary cell cultures, (2) clonal cell lines, and (3) neural stem cells. The main advantage of using primary cell cultures is that they retain the morphological, neurochemical, and electrophysiological properties of neurons *in situ*[2]. However, the disadvantages of primary cell cultures include (a) a limited life span, (b) increased genetic variability between model systems and cultures, (c) mixture of different neuronal populations in each preparation, as well as (d) high resource requirements [2]. Neural stem cells have the ability for self renewal and generating multiple cell types representing different parts of the nervous system; moreover, they can be derived from humans. However, as with primary cultures, the stem cells require animal use resulting in high resource requirements and regulatory issues. After neural stem cells have reached the end of their short variable life span, a new line has to be generated and then characterized before use [2,32]; this is neither time nor cost effective.

A clonal cell line is defined as a population of cells that originated from a single source and can be maintained in culture for an extended period of time. A clonal cell culture has a number of advantages that make them useful as *in vitro* models: easy to obtain; relatively easy to grow; divide rapidly; and can be continuously subcultured for a relatively high number of passages to provide a large number of cells in a short period of time [2]. The clonal M17 neuroblastoma cell line used in this study has the characteristics described above as well as the ability to become differentiated into a neuroblastic (N) cell when cultured in the presence of RA for several days [11,14]. These properties make the M17 cell line a good *in vitro* cell model for mechanistic and neurotoxicity testing. However, the functional changes in M17 cells due to RA differentiation have not been thoroughly characterized. A very relevant question is why do we need a differentiated neuronal model for neurobiology studies. The answer is that most of the neuronal functions such as membrane excitability, ion channels, neurotransmitter release, endocyctotic and exocyctotic events etc. are characteristics of mature neurons and cannot be studied in an immature neuronal model.

Treatment with RA results in the progressive development of neuronal morphologies and formation of neuronal networks seen in a more mature neuronal culture over time. A commonly measured characteristic of differentiation is extension of neurites that are akin to the axons and dendrites of fully differentiated neurons [33]. These neuronal properties in RA differentiated M17 cells were evidenced by both light microscopic observations (Figure 1) and immunofluorescence staining (Figure 2B - D). Although differentiated M17 cells demonstrated evidence of maturing neuronal organization and properties, the functional verification of synaptic activity remains to be done.

Besides morphological characteristics, we also observed differences in expression of several neuron specific proteins between undifferentiated and differentiated M17 cells (Figure 4). The presence or levels of certain proteins can vary between immature and mature neurons. One such protein is neuron specific enolase (NSE) which is responsible for generating phosphopyruvate hydratase that participates in glycolosis/gluconeogenesis; the levels of NSE increase as the neurons mature. While undifferentiated M17 cells do express NSE, its level increases due to differentiation. The formation of neurite-like processes as a part of synaptic organization and activity can be further characterized with the differential expression of the neurofilament proteins, NF-M, and –H that help form the neurofibrils within axons [33]. Developing neurons generally do not express either of these neurofilament proteins until they become post-mitotic, which is fairly late in development. Another neurofilament subunit, vimentin, decreases as neurons mature [34]. We were able to detect vimentin (Figure 4C) and the neurofilament proteins -M as well as -H (data not shown). We observed a decreased level of vimentin, whereas neurofilaments H and M increased due to differentiation. This might be an indication that under the conditions used, M17 cells could be in an early stage of maturation. This hypothesis is supported by the wide-spread expression of the immature neuronal marker β3-tubulin and the accumulation of synapsin-1/2 at the tip of the growth cone (Figure 3)*.* The presence of synapsin within the growth cone is consistent with studies suggesting an axonogenic role during neurite extension and branching, which is a early aspect of neuronal maturation [35,36]. The levels and localization of these developmentally staged proteins are anticipated to further change during prolonged culture in the presence of RA.

Since M17 cells are multipotential with regard to neuronal enzyme expression, we looked at the effects of RA differentiation on the expression of the main isoforms of acetylcholine (ACh) receptors (M1 mAChR, nAChR – α7) and choline acetyltransferase (ChAT) to determine the type of neurons that RA differentiated M17 cells could be. In Figure 5, nAChR-α7 was the only one of the mentioned proteins that was able to be detected. This indicates that the RA differentiated M17 cells are not cholinergic but would most likely be involved in post- and pre-synaptic excitation in the brain and not post-ganglion nerves in the CNS or exocrine glands [37,38].

The differential expression of other neuronal proteins than those previously described, expression of voltage-gated Ca^2+^ channels and ionotropic receptors, which ultimately lead to an increase in neurotransmitter release, can be used to confirm neuronal characteristics and neuroexocytosis. The presence of SNAP-25 and synapsin are indicative of the potential to form functioning pre-synaptic compartments that mediate synaptic vesicle fusion with the pre-synaptic membrane and neurotransmitter release under depolarizing conditions. Although immunoblot demonstrated that overall synapsin expression in M17 cells does not significantly change after differentiation with RA (Figure 4B), synapsin-1/2 becomes distributed along processes, with a punctuate appearance (Figure 2 and supporting Additional file 1: Figure S1) and within the growth cone during neuritogenesis (Figure 3)*.* SNAP-25 is a major component of the SNARE complex that is required for the fusion of vesicle to the cell membrane for the exocytosis of neurotransmitters. A two fold increase in the level of SNAP-25 (Figure 4A) was observed. This increase in SNAP-25 level may correlate with the significant (p<0.01) increase in KCl stimulated [^3^H] glycine release seen in differentiated M17 cells (Figure 6). The increase in the stimulated release as shown in Figure 6 doesn’t look impressive; however, it is quite marked because we are comparing the fraction of the total pool of [^3^H] glycine that is released in differentiated cells vs. undifferentiated cells. As mentioned earlier, we studied [^3^H] glycine release because this assay has been utilized successfully in assessment of neurotoxicity in cell culture models [15-18]. Others have studied glutamate release and glutamate induced excitotoxicity in M17 cells [12] and as such these cells could be suitable to study glutamate neurotoxicity. Since M17 cells have been reported to have a poor GABAergic property [13] these cells might not be a suitable model for GABA studies. In this report we demonstrated that a representative neurotransmitter function is enhanced in differentiated M17 cells compared to immature cells.

For functional neuroexocytosis, neurons need both the ability to form the SNARE complex and to have functional voltage-gated Ca^2+^ channels. The ability of Ca^2+^ and other ions to move across the cell membrane is necessary for excitation and signal transmission between neurons. Therefore, we studied the uptake of Ca^2+^ in both undifferentiated and differentiated M17 cells. There was no increase in the uptake of radiolabeled ^45^Ca^2+^ using varying concentrations of KCl in undifferentiated M17 cells (Figure 7A); a strong increase in the uptake of radiolabeled ^45^Ca^2+^ was observed with RA differentiation of M17 cells with a maximum opening of voltage-gated Ca^2+^ channels at 25 mM KCl. The presence of both N and P/Q type Ca^2+^ channels was indicated by the 50 – 60% reduction in Ca^2+^ uptake when conotoxin GVIA (N-type blocker) (Figure 7D) or agatoxin IVA (P/Q blocker) (Figure 7E) were applied to the culture. Using NNC 55–0396, only a small amount of T-type Ca^2+^ channels were detected (Figure 7C); however, the assay may not be sensitive enough to pick up the small change in intracellular Ca^2+^ concentration due to the small unitary conductance of T-type Ca^2+^ channels. Since neuroexocytosis is Ca^2+^ dependent, the lack of functional voltage-gated Ca^2+^ channels in undifferentiated M17 cells is detrimental for its use as a cell model for neurotoxicity research. The treatment of M17 with RA for a minimum of 72 hrs may be essential for functional neuronal cultures. It has been postulated that neuronal functions are cell maturation dependent [17].

In toxicity studies, it is important to look at both morphological and functional changes to determine toxicological mechanisms. It has been shown here as well as in other studies that maturation of neuronal cultures is very important when studying the effects of toxicants [17]. It is known that intracellular Ca^2+^ is highly regulated and involved in normal cell functions and in toxicological mechanisms. The lack of voltage-gated Ca^2+^ channel expression in undifferentiated M17 cells could limit their use as a neurotoxicity model. This is supported by our observation that differentiation of M17 cells with RA was required to see the changes in [Ca^2+^]_i_ following exposure to CG. The [Ca^2+^]_i_ decrease due to CG is a toxicant response in neuronal cells that can lead to apoptosis and death of neurons [39,40]. Acquisition in voltage-gated Ca^2+^ channels in differentiated neurons may be a prerequisite for studying neurotoxicity due to chemicals other than CG.

## Conclusion

The results reported here show that the human neuroblastoma BE(2)-M17 cells need to be treated with RA to become differentiated into mature neurons and to exhibit functional neuroexocytosis. Differentiation with RA induces M17 cells to undergo morphological differentiation and synaptic maturation. The apparent formation of neural networks, the presence and function of SNARE proteins and voltage-gated Ca2+ channels are necessary for functional neuroexocytosis. Our results showing the presence of these characteristics supports the use of differentiated M17 cells as a cell model for neurobiology and/or neurotoxicity research.

## Abbreviations

M17: BE(2)-M17 cell line; Ca2+: Calcium; RA: Trans-retinoic acid; NSE: Neuron specific enolase; SNAP-25: Synaptosomal-associated protein 25; nAChR-α7: Nicotinic acetylcholine receptor α7; CG: Phosgene; Pb: Lead; OP: Organophosphorus; S: Substrate-adherent; N: Neuroblastic; I: Intermediate; NF-H: Neurofilamentin heavy chain; NF-M: Neurofilamentin medium chain; NF-L: Neurofilamentin light chain; PBSS: 0.1% saponin in PBS and blocked with 3% BSA; Ach: Acetylcholine; M1 mAChR: M1 muscarinic acetylcholine receptor; ChAT: Choline acetyltransferase; ECF: Enhanced chemifluorescent; [Ca2+]i: Intracellular free Ca^2+^ concentration; CNS: Central nervous system; SNARE: Soluble NSF attachment protein receptor.

## Competing interests

The authors declare that they have no competing interests, financial or non-financial.

## Authors’ contributions

Participated in research design: DA, BK, KH, PMcNutt, RR. Conducted experiments: DA, BK, JP, BB, KH. Contributed new reagents or analytic tools: N/A. Performed data analysis: DA, BK, JP, KH. Wrote or contributed to the writing of the manuscript: DA, BK, KH, PMcNutt, RR. Other: DA, BK, RR acquired funding for the research. All authors read and approved the final manuscript.

## Supplementary Material

Additional file 1: Figure S1 Split confocal image of synapsin-1/2 and β3-tubulin expression in RA-induced M17 cells at 120 h. M17 neuroblastoma cells were grown on cover slips. Cells were fixed, stained, and immunofluorescent images were taken (63X). Synapsin-1/2 (green), β3-tubulin (red) and nuclei (blue). Split panels diffuse synapsin expression in cell body; with punctuate expression apparent in elongated neurites. Click here for file
