# Supplementary figures and images for: Morphological and functional differentiation in BE(2)-M17 human neuroblastoma cells by treatment with Trans-retinoic acid
Source: BMC Neurosci. 2013 Apr 18;14:49. doi: 10.1186/1471-2202-14-49 (PMC3639069; doi:10.1186/1471-2202-14-49)

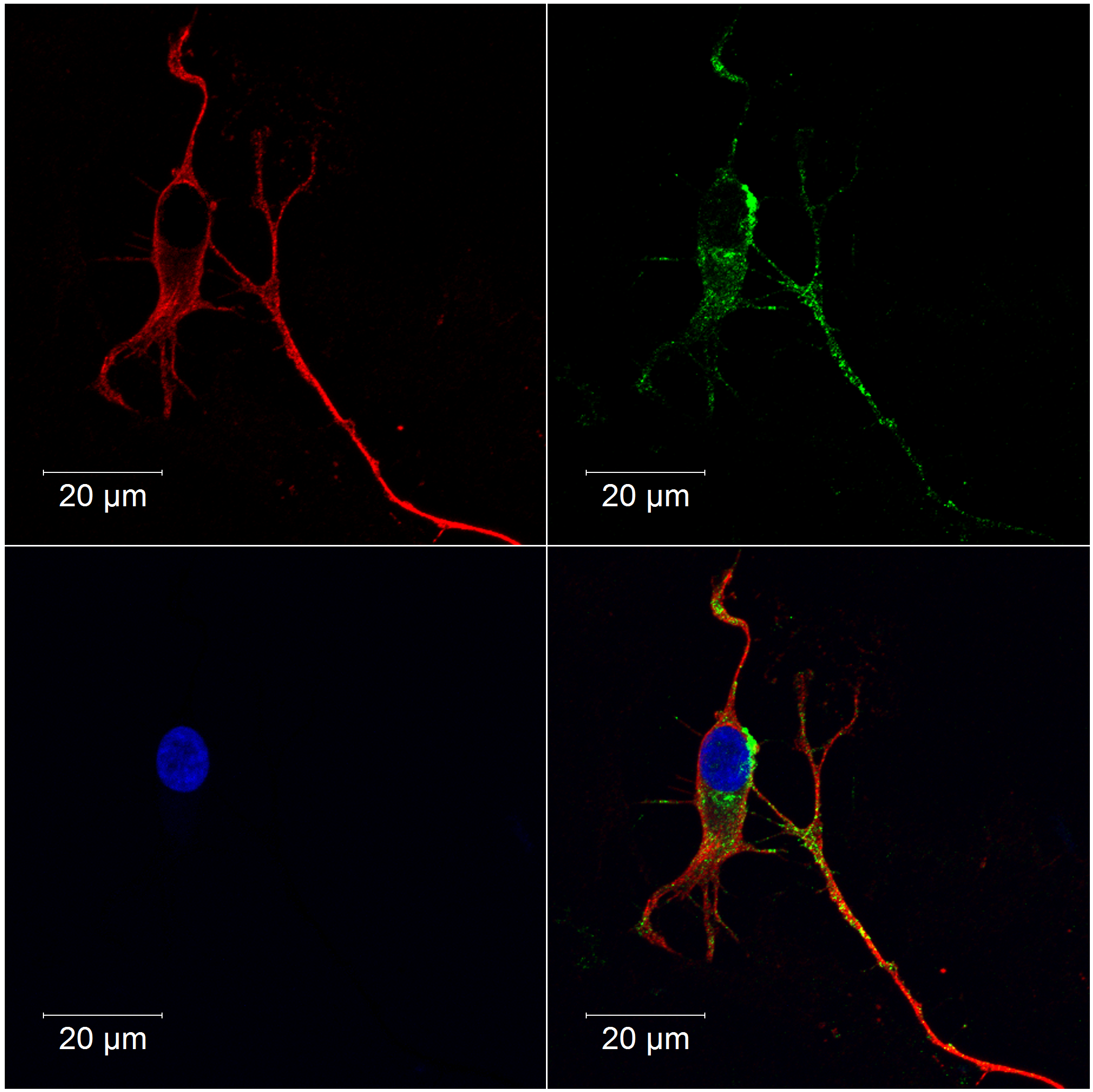

Supplement: Additional file 1: Figure S1 — Split confocal image of synapsin-1/2 and β3-tubulin expression in RA-induced M17 cells at 120 h. M17 neuroblastoma cells were grown on cover slips. Cells were fixed, stained, and immunofluorescent images were taken (63X). Synapsin-1/2 (green), β3-tubulin (red) and nuclei (blue). Split panels diffuse synapsin expression in cell body; with punctuate expression apparent in elongated neurites. [file 1471-2202-14-49-S1.tiff]
